# The construction of a prognostic model of cervical cancer based on four immune-related LncRNAs and an exploration of the correlations between the model and oxidative stress

**DOI:** 10.3389/fphar.2023.1234181

**Published:** 2023-09-21

**Authors:** Xuefeng Lv, Yanyan Jia, Jinpeng Li, Shu Deng, Enwu Yuan

**Affiliations:** ^1^ Department of Laboratory Medicine, The Third Affiliated Hospital of Zhengzhou University, Zhengzhou, Henan, China; ^2^ Department of Gynecology and Obstetrics, The First Affiliated Hospital of Zhengzhou University, Zhengzhou, Henan, China

**Keywords:** cervical cancer, immune-related lncRNAs, prognosis, immunotherapy, oxidative stress

## Abstract

**Introduction:** The immune-related lncRNAs (IRLs) are critical for the development of cervical cancer (CC), but it is still unclear how exactly ILRs contribute to CC. In this study, we aimed to examine the relationship between IRL and CC in detail.

**Methods:** First, the RNAseq data and clinical data of CC patients were collected from The Cancer Genome Atlas (TCGA) database, along with the immune genes from the Import database. We used univariate cox and least absolute shrinkage and selection operator (lasso) to obtain IRLs for prediction after screening the variables. According to the expression levels and risk coefficients of IRLs, the riskscore were calculated. We analyzed the relationship between the model and oxidative stress. We stratified the risk model into two as the high and low-risk groups. We also evaluated the survival differences, immune cell differences, immunotherapeutic response differences, and drug sensitivity differences between the risk groups. Finally, the genes in the model were experimentally validated.

**Results:** Based on the above analyses, we further selected four IRLs (TFAP2A.AS1, AP000911.1, AL133215.2, and LINC02078) to construct the risk model. The model was associated with oxidative-stress-related genes, especially SOD2 and OGG1. Patients in the high-risk group had a lower overall survival than those in the low-risk group. Riskscore was positively correlated with resting mast cells, neutrophils, and CD8+ T-cells. Patients in the low-risk group showed a greater sensitivity to immunosuppression therapy. In addition, we found that patients with the PIK3CA mutation were more sensitive to chemotherapeutic agents such as dasatinib, afatinib, dinaciclib and pelitinib. The function of AL133215.2 was verified, which was consistent with previous findings, and AL133215.2 exerted a pro-tumorigenic effect. We also found that AL133215.2 was closely associated with oxidative-stress-related pathways.

**Discussion:** The results suggested that risk modeling might be useful for prognosticating patients with CC and opening up new routes for immunotherapy.

## Introduction

Globally, CC is the fourth leading cause of cancer-related deaths among women (Sawaya et al., 2019; Wen et al., 2020). Although the screening for CC and human papillomavirus (HPV) vaccination programs has been developed, the number of newly-diagnosed CC cases is on the rise, implying that CC remains a major public health concern (Arbyn et al., 2020). Surgery, radiotherapy and chemotherapy are the three common treatments for patients with CC, however, its 5-year survival rate remains unsatisfactory, owing to recurrence, metastasis and drug resistance (Sol et al., 2009; Kumar et al., 2018; Marquina et al., 2018). The progression and treatment of CC are influenced by the immune system (Chen et al., 2019); hence, immunotherapy is an effective treatment option for patients with CC. With the use of immune checkpoint inhibitors for cancers, great progress has been made in immune-targeted therapies for CC. Immunotherapies comprising anti-CTLA4 and anti-PD1 drugs are effective against CC(Drews et al., 2019). However, the consistently-low positive immunotherapeutic response limits the development and application of immunotherapies for patients with CC (Chung et al., 2019). It is therefore crucial to identify new therapeutic targets and biomarkers for the early diagnosis and prognosis of CC. Non-coding RNAs with more than 200 nucleotides are called long-stranded non-coding RNAs (lncRNAs), which can be involved in post-transcriptional modifications (Kung et al., 2013)and play a key role in processes such as antigen presentation, cancer immunity as well as immune cell infiltration (Denaro et al., 2019; Zhang L. et al., 2020). The lncRNA CamK-A, for example, is highly expressed in several human cancer types and can regulate the Ca2+-signaling-mediated remodeling of the tumor microenvironment (Sang et al., 2018). In addition, the overexpression of HLA-F-AS1 in colorectal cancer cells suppresses miR-375 and promotes the expression of PFN1, thereby exacerbating tumorigenesis (Zhang et al., 2021). LncRNAs can influence the response of patients with cancers to immunotherapies and the tumor microenvironment (Zhang Y. et al., 2020). However, little has been reported about the action mechanism of IRLs in patients with CC. Oxidative stress is involved in the development and progression of many diseases, including cancers (Valko et al., 2007), which is mainly because it can cause inflammation and thus affect cancer development (Reuter et al., 2010). Oxidative stress also plays an important role in CC. It has been shown that triflavin can induce apoptosis by regulating oxidative stress, thereby inhibiting cervical carcinogenesis (Zhu et al., 2021). In addition, oxidative stress is critical in lipid peroxidation, which has a positive effect on the elimination of HPV-related cancers (Cruz-Gregorio et al., 2021). Therefore, it is necessary to discover a new IRL as a potential marker of CC and explore its associations with oxidative stress.

Using the TCGA database and RNA sequencing data, we identified IRLs and established a 4-IRL risk model through the Lasso method. We also explored the potential links between the risk model and oxidative stress. Subsequently, we examined several clinical characteristics of patients with CC that were associated with the model. Additionally, the correlations of the IPS with single nucleotide polymorphism (SNP) mutations, copy number variations (CNVs) and immune cell infiltration were also analyzed. An analysis of drug sensitivity was conducted to improve drug treatment. Overall, these findings may provide a strategy for the prognostic prediction of patients with CC, along with the identification and development of immune-related treatment targets.

## Materials and methods

### The acquisition of data and the screening of immune-related lncRNAs

The transcriptomic and clinical data (detailed information about the demographic characteristics of the patients in Supplementary Table S3) on CC (normal = 3,tumor = 306) was obtained from the TCGA database (https://tcga-data.nci.nih.gov/), and the immune-related genes were accessed from the Import database (https://www.immport.org/). Count values of raw data were converted to transcripts per kilobase million (TPM) values for subsequent analyses; count values were used only to identify the differential genes. To identify differentially-expressed lncRNAs, we compared different gene expressions between normal and tumor samples with a threshold of |log2 FC (Log2 Fold Change)| > 2 and FDR (false discovery rate) < 0.01. IRLs were obtained based on the relationship between the expression of lncRNAs and immune genes using the Person correlation test (correlation coefficients >0.6). By taking the intersections of DElncRNAs and IRLs, the relevant IRLs were obtained.

### The construction and validation of risk model

Machine learning is widely used in applications such as nearest neighbour search in large-scale data (Yan et al., 2021), dimensionality reduction of features, etc., We filtered the significant prognostic lncRNAs with *p* < 0.05 through univariate Cox analysis and identified the final lncRNAs using lasso regression analysis. In order to create a prognostic risk model, we used the coefficients obtained from the lasso to calculate the riskscore of each patient with CC. The calculation was as follows:

$$riskScore=\sum_{i=1}^{n}Coefi*xi$$

The coefficient is Coefi, while xi is the count value of each DElncRNA. Based on the median riskscore (The advantages of the median are that it makes full use of all data information to reflect the centralized trend of a group of data, is not affected by extreme data, and is easy to find. It can clarify the middle level and is less affected by extreme data. Disadvantages: It is easily affected by extreme values), patients were divided into two groups, namely, the high-risk and the low-risk group. Furthermore, based on the survival duration of patients, a Kaplan-Meier analysis was performed to evaluate their prognostic value. Model-based receiver operating characteristic (ROC) curves were plotted for the first, third and fifth year; based on the survival time of patients, Kaplan-Meier analyses were performed, and survival curves were used to display the risk model results.

### The acquisition of oxidative-stress-related genes

We collected several common oxidative-stress-related genes from published studies, including SOD1, SOD2 (Zelko et al., 2002), PON1(Teranishi et al., 2012), NOS3(Katkam et al., 2018), UCP2(Hu et al., 2019), GSR (Couto et al., 2016), GPX1 (Teranishi et al., 2012) and GSTM1(Cadoni et al., 2006). 8-hydroxy-2 deoxyguanosine (8-OHDG) is known as a key marker of oxidative stress (Reuter et al., 2010) and we collected 8-OHDG-related genes from GeneCards (https://www.genecards.org/) and obtained gene enrichment pathways using ClueGO. A protein-protein interaction network was then obtained through the String website (https://cn.string-db.org/). Four methods based on the naximal clique centrality (MCC), the density of maximum neighborhood component (DMNC), the maximum neighborhood component (MNC) and degree of cytoHubba were used to screen key genes, the top 10 of which were crossed. Correlations between the risk model and oxidative-stress-related genes were analyzed using the Spearman test.

### The correlation between the risk model and clinical characteristics

The correlations between the model and the age, grade, clinical stage as well as TNM stage of patients with CC were assessed using the Chi-square test.

### The correlation between targeted therapeutic markers and the risk model

Microsatellite instability (MSI), tumor mutational burden (TMB) and homologous recombination deficiency (HRD) are common molecular characteristics of genomic instability, which are validated biomarkers for targeted therapies (26). We performed a Kaplan-Meier analysis for TMB, MSI and HRD to assess their prognostic values. A Chi-square test was also used to evaluate their associations with the risk model.

### Gene mutations and copy number variants

We downloaded the data on SNPs and CNVs of patients with CC from TCGA and UCSC databases. The SNPs and CNVs were visualized using circos (http://circos.ca) and R. The focus was on the demonstration of their locations on the chromosomes where the genes were present in the model. Significantly-mutated genes (*p* < 0.05) and gene mutation interactions between the high- and low-risk group were analyzed using the MAFTOOLS software. In both analyses, only the genes mutating more than 10 times in at least one group were considered, whose expression was probed using GEPIA. A statistical test for significant mutation rates was performed using a one-sided z-test. Copy number alterations among patients with CC were analyzed with GISTIC 2.0 (Mermel et al., 2011). The copy number gistic score, together with the percentage of patients in both risk groups, was also analyzed.

### The infiltration of immune cells

We obtained most of our immune cell data from XCELL (Aran et al., 2017), EPIC(Racle et al., 2017) and CIBERSORT (Newman et al., 2015). Next, the immune cell infiltration was quantified using ssGSEA for subsequent analyses (Barbie et al., 2009; Bindea et al., 2013). Further analyses were conducted on the correlations between immune cell types and immune cell content of the risk groups. Based on a Pearson correlation analysis, we analyzed how immune cells and IRLs interacted.

### The prediction of immunotherapeutic response

We used unsupervised subclass mapping methods (https://cloud.genepattern.org/gp) to predict the responses of different risk groups to immunotherapies (Lu et al., 2019).

### Drugs with differential sensitivities in high- and low-risk groups

PIK3CA mutations are more common in CC (Xiang et al., 2015). As a result, mutations in the PIK3CA gene can be used as a target biomarker for patients with CC. We segregated the patients with CC carrying PIK3CA mutations. After downloading data on drug sensitivity AUC value from the Cancer Therapeutics Response Portal (CTRP2.0), Profiling Relative Inhibition Simultaneously in Mixtures (PRISM) repurposing dataset and the Cancer Cell Line Encyclopedia (CCLE) expression profile, we performed a differential drug response analysis on patients in both risk groups. Spearman correlation analysis (*r* < −0.30 for CTRP; *r* < −0.35 for PRISM) was used to screen compounds with negative correlation coefficients (Yang et al., 2021).

### Experimental verification

A total of 22 pairs of cancerous and non-tumorous tissues were collected from patients with CC at the First Affiliated Hospital of Zhengzhou University. This study was approved by the Ethics Committee of the First Affiliated Hospital of Zhengzhou University (Ethics Number: 2022-KY-0093-002), and an informed consent was obtained from patients. qRT-PCR was used to determine the expression of lncRNAs through the risk model. Primers for TFAP2A-AS1, AP000911.1, AL133215.2 and LINC02078 were designed using primer 5.0 (Supplementary Table S1). Total RNA was extracted with a trizol (CWBIO, China), and the first strand of cDNA was synthesized using a reverse transcription kit (Takara, Kyoto, Japan). Finally, cDNA was quantified through qRT-PCR using SYBR green master mix (Vazyme, China). GAPDH was used as an internal reference for calibration. The 2^−ΔΔCT^ method was chosen to calculate the relative expression of lncRNAs. Cellular functional assays were performed for AL133215.2, which was knocked down in the HeLa and SiHa cell line via transfection. Cells transfected with siRNA and controls were stained with an Annexin V-FITC apoptosis detection kit (Beyotime, Shanghai, China). The stained cells were then analyzed through flow cytometry. Relative cell viability was monitored 24, 48, 72, and 96 h after transfection using cell counting kit-8 (CCK-8, Beyotime, Shanghai, China).

### Gene set variation analysis (GSVA) and gene set enrichment analysis (GSEA)

We used GSVA to analyze the 50 hallmark pathways described in the molecular signature database (Subramanian et al., 2005; Hänzelmann et al., 2013). Next, we used a limma package to obtain pathways that differed significantly between patients in the high- and low-risk group. A GSEA (Subramanian et al., 2005) was conducted for both risk groups, and we selected significantly-enriched pathways based on *p*-values and FDR q-values that were below 0.05 and 0.25 respectively. We obtained the previously-reported gene sets related to immunotherapy from Hu et al. (2021). In addition to the immune-related gene sets we collected, gene ontology (GO) pathways associated with oxidative stress were also enriched using GSVA. Finally, we examined the associations between genes in the model and the enrichment scores.

### Developing a predictive nomogram

The nomogram-integrated factors including the risk score, T, N, MSI. calibration curves and ROC were used to evaluate the accuracy and predictive ability of the nomogram; decision curve analysis (DCA) was used to evaluate the clinical effectiveness of the nomogram.

### Statistical methods

R version 4.4.1 was used to perform all statistical tests in this manuscript. The χ2 test was used for appropriate categorical data, and the two-sample Wilcoxon test was used for continuous data. Survival analyses were performed using the R package “survival”. Correlation analysis was performed using the Pearson correlation test. Statistical significance was defined as a *p*-value of less than 0.05 for all statistical analyses.

## Results

### The construction and validation of the risk assessment model

**FIGURE 1:**
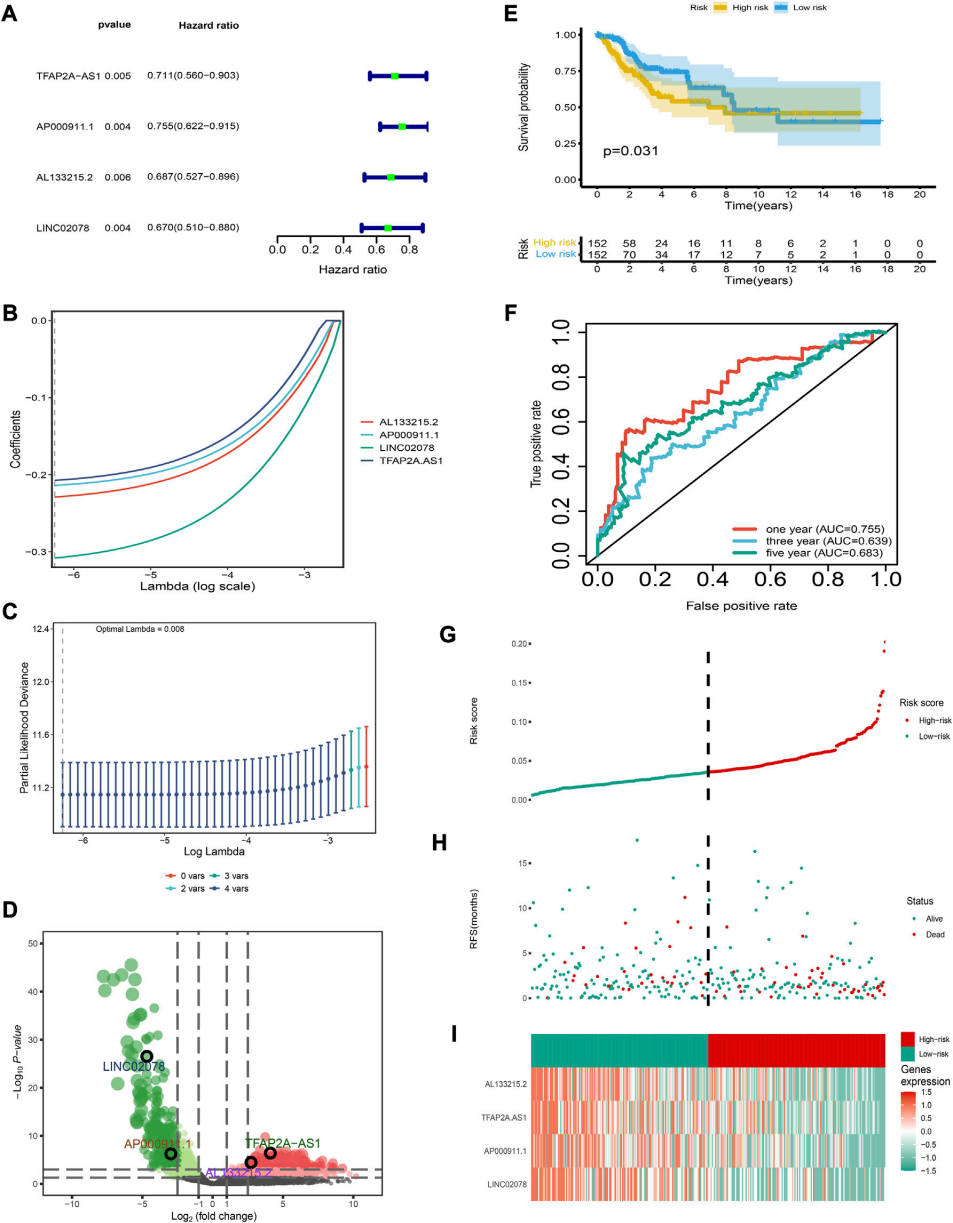
Establishment and verification of the risk model **(A)** Forest map for univariate Cox analysis. **(B)** LASSO coefficient distributions for four lncRNAs **(C)** Partial likelihood deviation of the LASSO coefficient distribution. Vertical dashed lines indicate lambda values. **(D)** Volcanic map of DEirlncRNAs. **(E)** The 3- and 5-year ROC curves for the risk model. **(F)** Patients in the low-risk group show longer survival as indicated by the Kaplan-Meier test. **(G–I)** Distribution of risk score, survival status, and molecular expression.

We obtained a total of 493 differentially-expressed lncRNAs; among them, 96 were immune-related lncRNAs (Supplementary Table S2). A total of four IRLs were identified and selected for risk modeling through univariate cox analysis (Figure 1A) and lasso analysis (Figures 1B, C); among them, TFAP2A-AS1 and AL133215.2 showed a significantly high expression, while LINC02078 and AP000911.1 had a significantly low expression in cancer tissues (Figure 1D). The risk score of each patient was computed as follows:

$$\text{Riskscore}=\text{TFAP}2A-\text{AS}1*\left(-0.207\right)+\text{AP}000911.1*\left(-0.214\right)+\text{AL}133215.2*\left(-0.229\right)+\text{LINC}02078*\left(-0.308\right)$$

The risk model had a good clinical predictive power, with a ROC value of 0.763, 0.645 and 0.678 for 1-, 3- and 5-year survival respectively (Figure 1E). The C-index and IBS of the risk model were 0.918 and 0.035, respectively, which also show that the risk model had a good predictive performance (details of the calculation process were in Supplementary Material S1). Patients in the low-risk group had a higher overall survival rate (Figure 1F). Furthermore, we determined the distribution of risk scores, the survival statistics of patients in different risk categories and the expression characteristics of the four IRLs (Figures 1G–I). As is shown in the graph, the low-risk patients showed an overexpression of these four protective lncRNAs.

### The relationship between the risk model and oxidative stress-related genes

**FIGURE 2:**
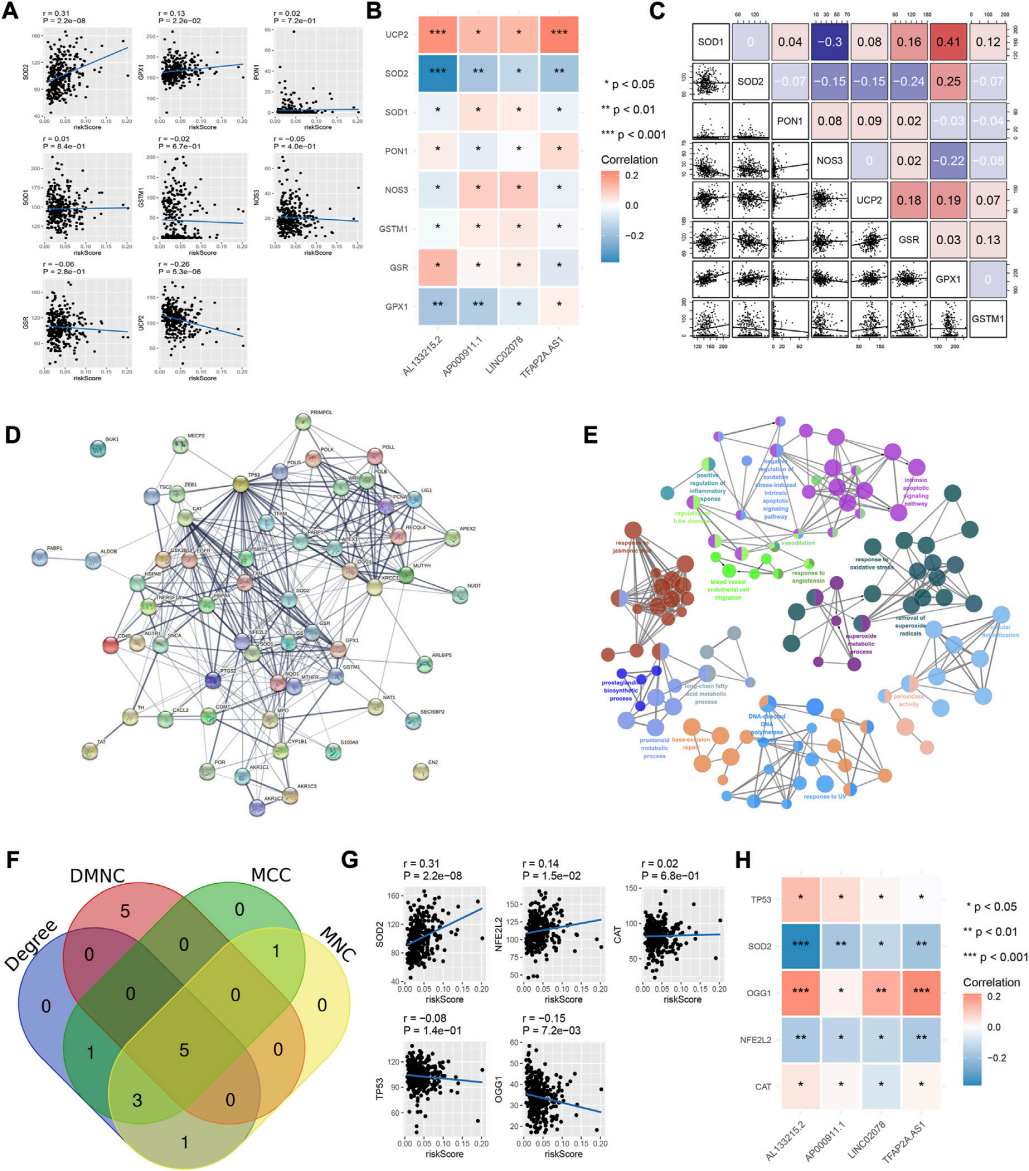
Relationship between risk model and oxidative stress-related genes. **(A)** Correlation of risk models and oxidative stress-related genes. **(B)** Correlation between the four IRLs and oxidative stress-related genes. **(C)** Correlation between oxidative stress-related genes. **(D)** Protein-protein interaction network of 8- OHDG-related genes. **(E)** Enrichment pathways of 8- OHDG-related genes. **(F)** Intersection genes of MCC, DMNC, MNC, and Degree. **(G)** Correlation of risk model and key genes **(H)** Correlation of 4 IRLs and key genes.

Among the eight common oxidative stress-related genes, the risk model was positively correlated with SOD2 while negatively correlated with UCP2 (Figure 2A). One of the four lncRNAs, AL133215.2, correlated significantly positively with SOD2 while negatively with UCP2; another was TFAP2A-AS1, which correlated significantly positively with UCP2. (Figure 2B). There was a significant positive correlation between SOD1 and GPX1 among the eight genes related to oxidative stress (Figure 2C). We obtained 62 8-OHDG-related genes from GeneCards, and the protein interaction network showed more interactions among TP53, OGG1, SOD2, CAT and other proteins (Figure 2D). The pathway enrichment results showed that 8-OHDG-related genes were significantly enriched in the negative regulation of oxidative stress-induced intrinsic apoptotic signaling pathways, responding to oxidative stress and other pathways (Figure 2E). Five key 8-OHDG-related genes were obtained through MCC, DMNC, MNC and Degree, namely, SOD2, OGG1, TP53, NFE2L2 and CAT (Figure 2F). The risk model and OGG1 were significantly negatively correlated (Figure 2G), and AL133215.2, TFAP2A-AS1 as well as OGG1 were significantly positively correlated (Figure 2H).

### Differences in clinical characteristics among risk groups

**FIGURE 3:**
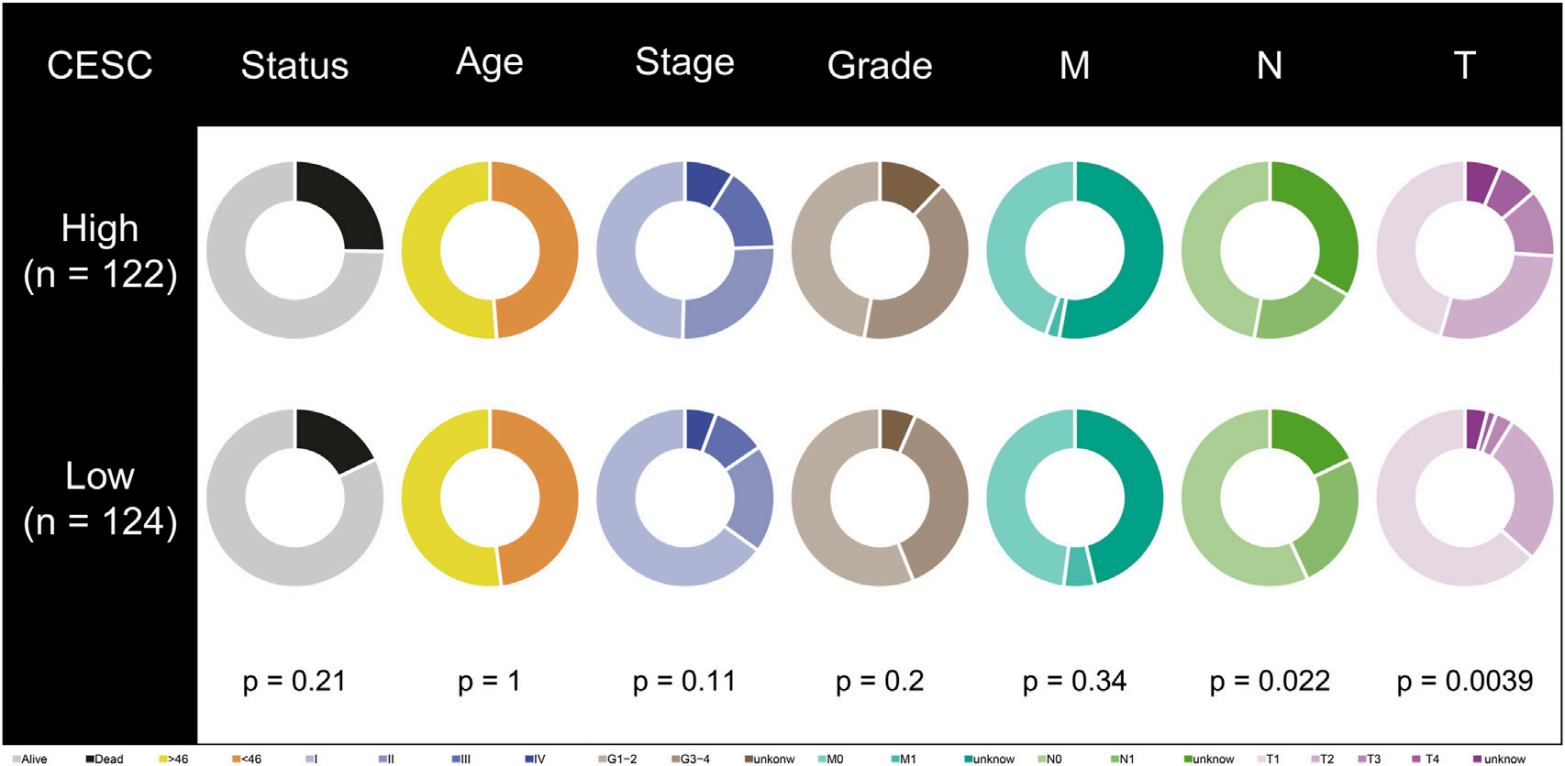
Correlation between risk model and clinical characteristics. T-stage and N-stage are different in high- and low-risk groups.

Among all clinical characteristics, N and T stage were significantly associated with the risk model (Figure 3). More patients in the low-risk group were in Stage N0, more of whom in the high-risk group were in Stage T3 and T4. Perhaps this is one of the reasons for the shorter overall survival time of patients in the high-risk group.

### The correlation between targeted therapeutic markers and the model

**FIGURE 4:**
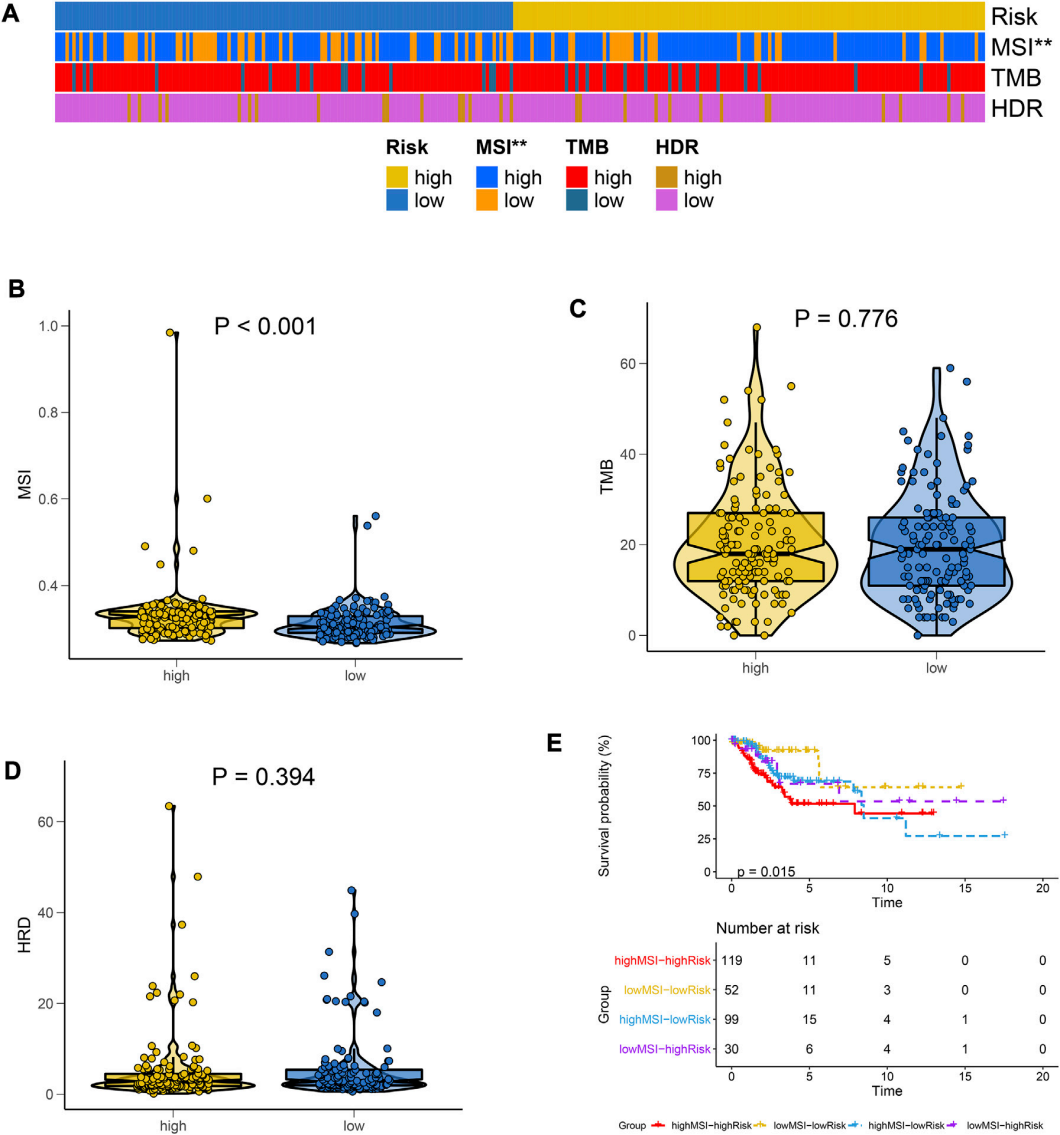
Differences in immunotherapy markers between the risk groups. **(A)** Heat maps for the distributions of MSI, TMB, and HRD in high- and low-risk groups**. (B–D)** The Violin plot for differences in expressions of MSI, TMB, and HRD between high- and low-risk groups. **(E)** MSI combined risk model was used to plot the survival curve.

Of the three markers, MSI and the risk groups were significantly correlated (Figure 4A). The high-risk group had a higher MSI value (Figure 4B). However, there was no significant difference between the two groups for TMB (Figure 4C) or HRD (Figure 4D). In a combined analysis on MSI and riskscore, highMSI-highRisk patients had a significantly shorter survival duration than lowMSI-lowRisk ones (Figure 4E). These results indicated that high-risk patients tended to have a higher MSI value, which contributed to a poorer prognosis.

### The mutation status of groups at a high and low risk

**FIGURE 5:**
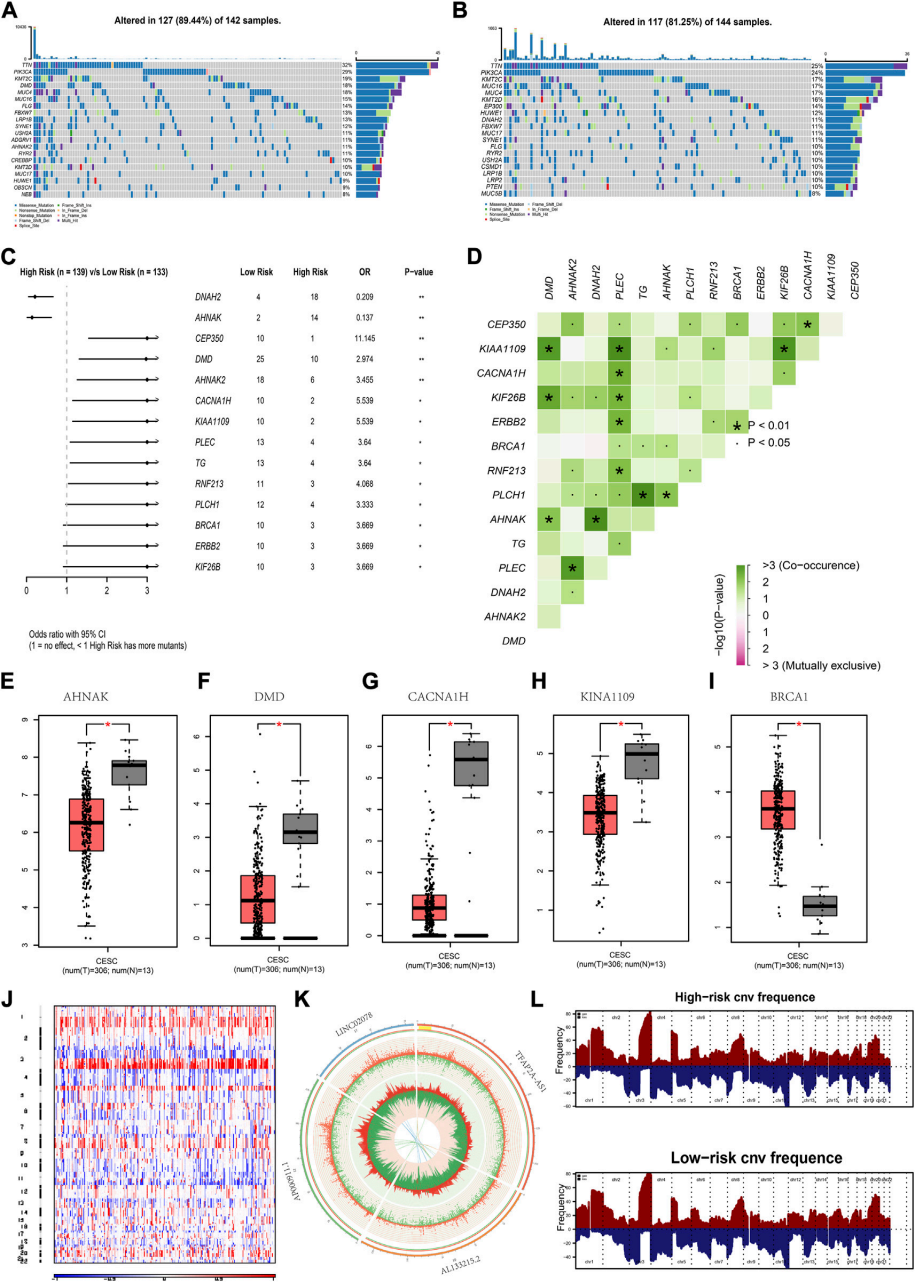
Mutations in high- and low-risk groups **(A,B)** Gene mutation waterfall map for low-risk group and high-risk group. **(C)** Forest map of the differentially mutated genes in the low-risk group and high-risk group. **(D)** Interaction of differentially mutated genes between the low-risk group and high-risk group. **(E–I)** Mutant genes showing expression differences between normal and CC patients. **(J)** Heat map of CNVs **(K)** Circle map of CNVs for the chromosomal location of genes of the risk model. **(L)** The chromosomal aberrations in high- and low-risk groups. *p < 0.05, **p < 0.01 indicated the statistical significance of data.

From the analysis, TTN, PIK3CA and KMT2C had a high mutation frequency in both high- and low-risk group (Figure 5A, B). There were two genes with a high frequency of mutations in high-risk patients, namely, DNAH2 and AHNAK. There were 12 genes with a high mutation frequency in low-risk patients, namely, DNAH2 and AHNAK (Figure 5C). These genes exhibited a significant co-occurrence (Figure 5D). AHNKA, DMD, CACAN1H, KINA1109 and BRCA1 were differentially expressed between the CC and normal group (Figures 5E–I). CNV results from patients with CC showed a greater significant increase in gene copy number on chromosomes 1 and 3 (Figure 5J). The CNV of chromosomes 6, 10, 11, and 17, where the four lncRNAs TFAP2A.AS1, AL133215.2, AP000911.1, and LINC02078 were located respectively, are shown in Figure 5K. As is shown in the figure, there was a higher gene copy number loss in these chromosomes. A significant similarity in the chromosomal aberrations of patients in the high- and low-risk group was also observed (Figure 5L).

### A comparison of the immune landscapes of high-risk and low-risk patients

**FIGURE 6:**
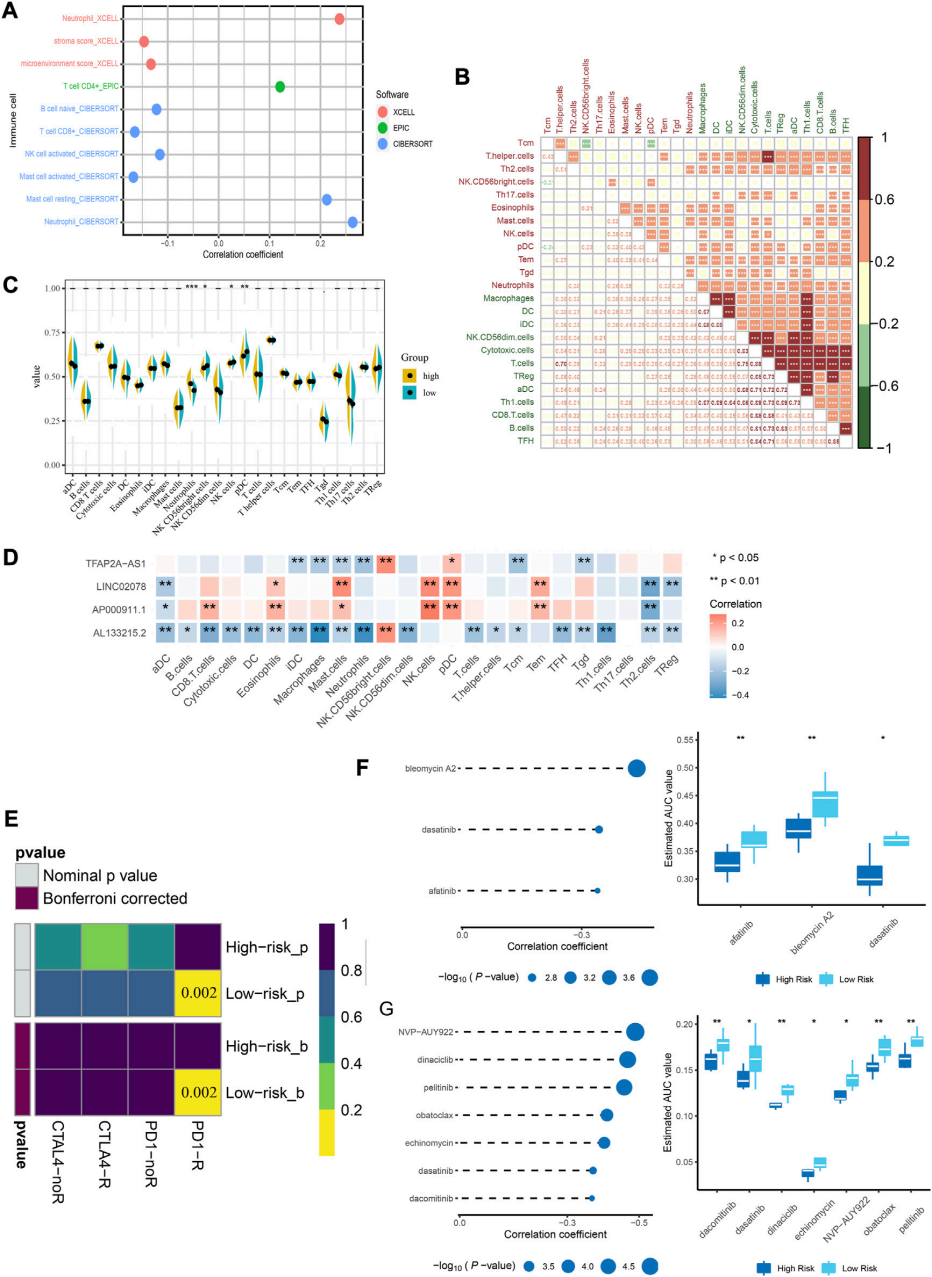
Immune infiltration landscape of CC and estimation of immunosuppressed genes using the risk model. **(A)** Correlation between immune cell types and riskscore. **(B)** Correlation matrix for the immune cells. **(C)** Comparison of the expressions of immune infiltrating cells in low- and high-risk groups. **(D)** Correlation between lncRNA and immune cells. **(E)** Submap analysis shows that patients in the low-risk group are more sensitive to PD-1 inhibitors. **(F)** Correlation analysis and drug response analysis for three differential drugs in CTRP. **(G)** Correlation analysis and drug response analysis for seven differential drugs in PRISM. *p < 0.05, **p < 0.01 indicated the statistical significance of data.

Among the immune cell types, assessed by several methods, a significant positive correlation was shown between neutrophils and riskscore (Figure 6A). When ssGSEA was used to quantify immune cells, most of them showed a strong positive relationship with each other (Figure 6B). Neutrophils, NK cells and pDCs were significantly different between the high- and low-risk group (Figure 6C). Among the four lncRNAs in the risk model, the expression of TFAP2A-AS1 and AL133215.2 was negatively correlated with that of the majority of immune cells, while LINC02078 and AP000911.1 showed opposite trends (Figure 6D). We used the submap analysis to compare the gene expression profiles of the defined high- and low-risk group with another dataset containing 47 melanoma patients who showed immunotherapeutic responses (Roh et al., 2017). Anti-PD-1 therapies were more likely to be effective for low-risk patients (Bonferroni corrected *p* = 0.015) (Figure 6E). We screened several drugs that showed sensitivity among low-risk patients, including bleomycin A2, dasatinib and afatinib in CTRP2.0 (Figure 6F) as well as NVP-AUY922, dinaciclib, pelitinib, obatoclax, echinomycin, dasatinib and dacomitinib in PRISM (Figure 6G). Based on these studies, we could develop individualized treatment plans for patients in different risk groups.

### The experimental validation of molecules in the model

**FIGURE 7:**
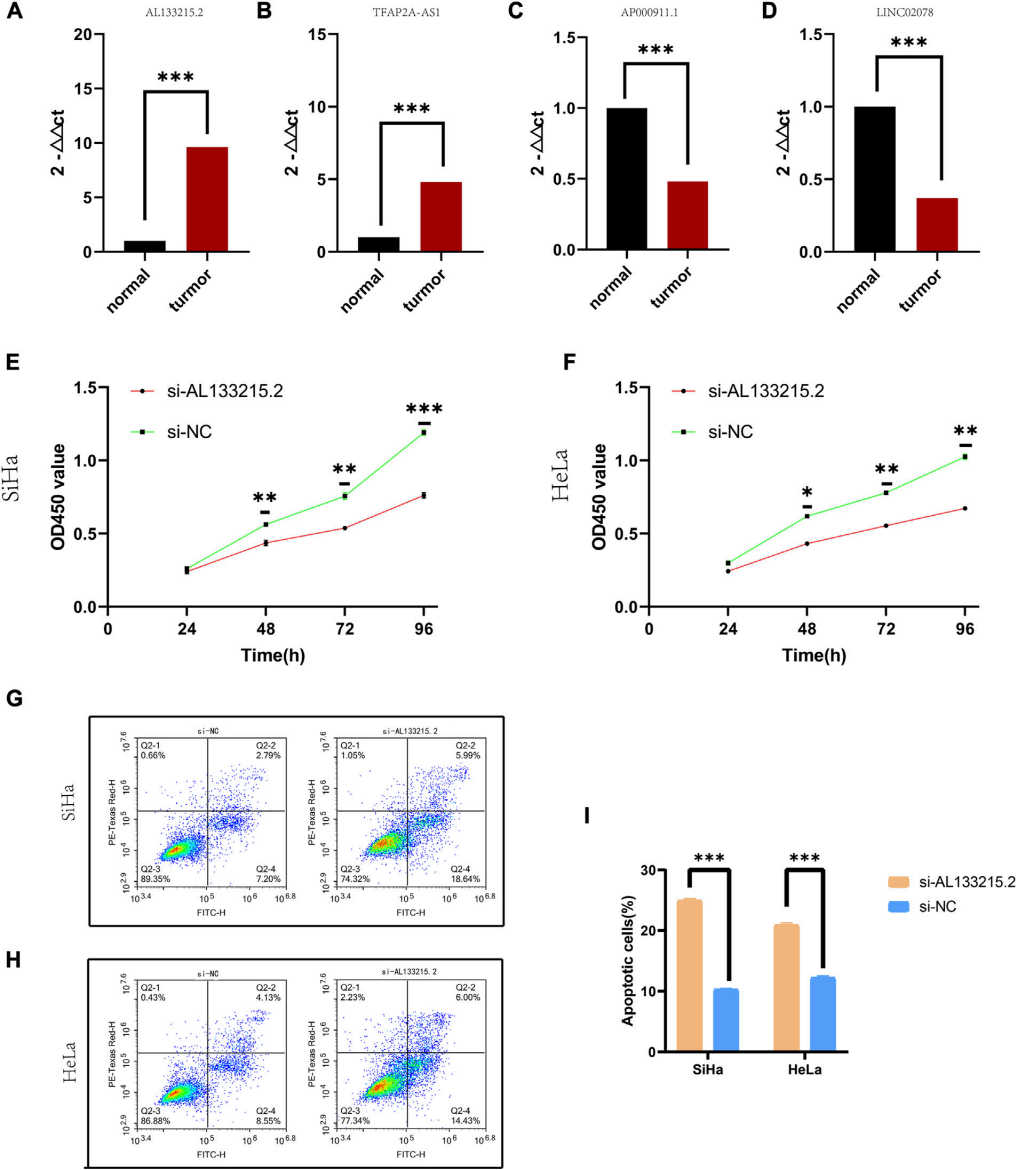
Experimental validation of genes in the risk model **(A–D)** qRT-PCR results for TFAP2A.AS1, AP000911.1, AL133215.2, and LINC02078. **(E)** CCK-8 results for knockdown of AL133215.2 in SiHa cell line. **(F)** CCK-8 results for knockdown of AL133215.2 in the HeLa cell line. **(G)** Apoptosis after knocking down AL133215.2 in SiHa cells **(H)** Apoptosis after knocking down AL133215.2 in HeLa cells **(I)** Histogram for apoptosis rates. *p < 0.05, **p < 0.01, ***p < 0.001 indicated the statistical significance of data.

The qRT-PCR assay indicated that TFAP2A-AS1 and AL133215.2 showed a significantly high expression in cancer tissues, while LINC02078 and AP000911.1 had a significantly low expression, which were consistent with our previous predictions (Figures 7A–D). Given the above analyses, we found a close association between AL133215.2 and oxidative-stress-related genes, so AL133215.2 was selected for experimental validation. CCK-8 assay showed that the proliferation of CC cells could be significantly inhibited by knocking down AL133215.2 (Figures 7E, F). We analyzed CC cells through flow cytometry and showed that apoptosis was significantly accelerated in both Siha and Hela cells after AL133215.2 knockdown (Figures 7G–I). These experimental results demonstrated the ability of AL133215.2 to promote the progression of CC.

### Pathway enrichment analysis

**FIGURE 8:**
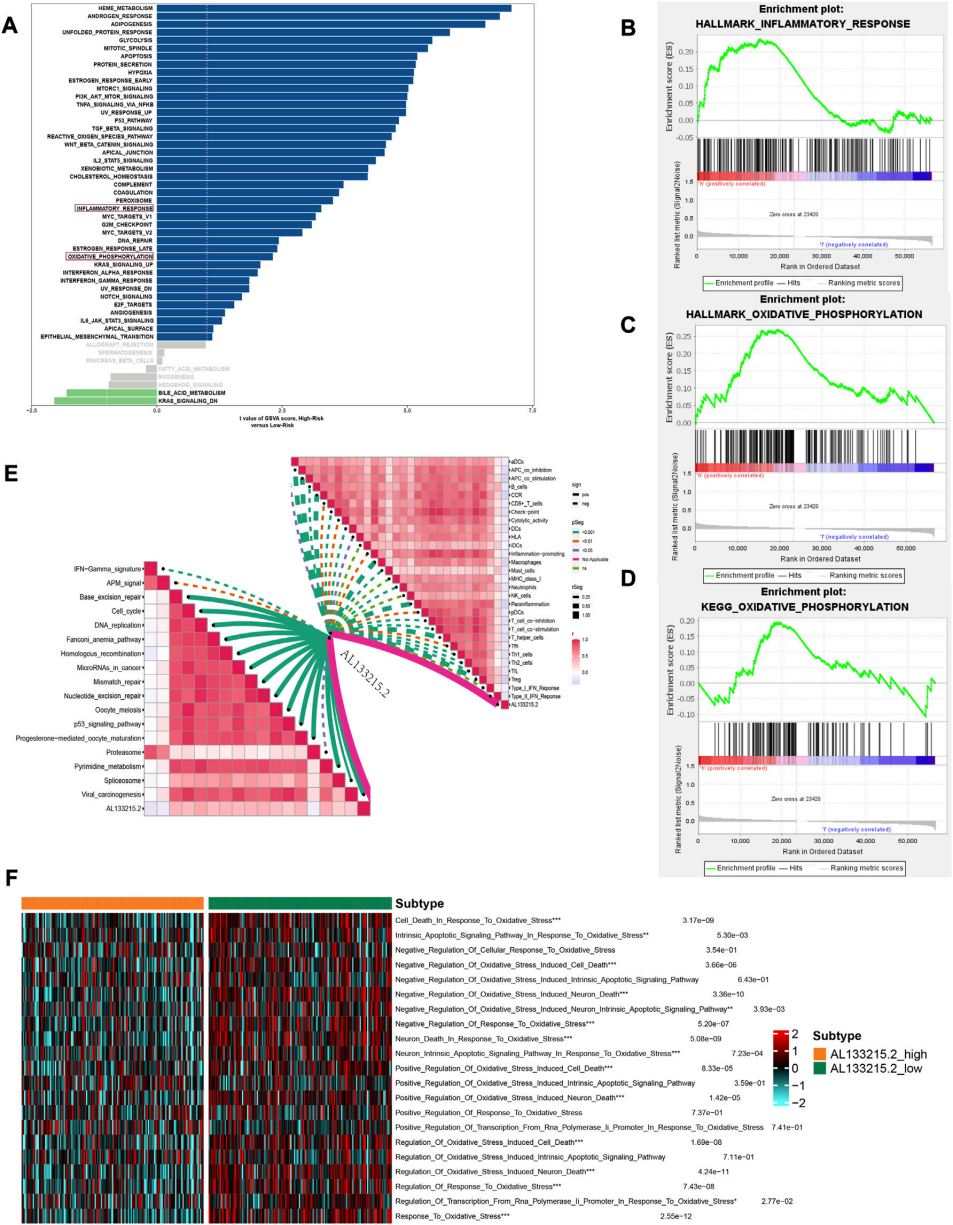
Gene set variation analysis and gene set enrichment analysis results. **(A)** Differential pathways between high- and low-risk groups**. (B)** Inflammatory_response in the HALLMARK gene set is significantly enriched in the high-risk group **(C)** Oxidative_phosphorylation in the HALLMARK gene set is significantly enriched in the high-risk group **(D)** Oxidative_phosphorylation in the KEGG gene set is significantly enriched in the high-risk group **(E)** Correlation of AL133215.2 with immune-related pathways **(F)** Differences in oxidative stress-related pathways in the high- and low-expression groups of AL133215.2. *p < 0.05, **p < 0.01, ***p < 0.001 indicated the statistical significance of data.

GSVA was performed on each patient to compare pathways between high- and low-risk group. Among high-risk patients, inflammatory response and oxidative phosphorylation were significantly enriched (Figure 8A). According to GSEA results of the HALLMARK and KEGG gene set, inflammatory response and oxidative phosphorylation were significantly enriched in high-risk individuals (Figures 8B–D). A positive correlation was found between AL133215.2 and most immunotherapeutic predictive pathways, while a negative one was seen in all immune precursor pathways (Figure 8E). The AL133215.2 low-expression group had a higher enrichment score in terms of the oxidative stress pathways (Figure 8F). This was consistent with our previous study, where the high-risk group corresponded to a lower expression of AL133215.2 and a more pronounced oxidative stress.

### Nomogram construction and evaluation

**FIGURE 9:**
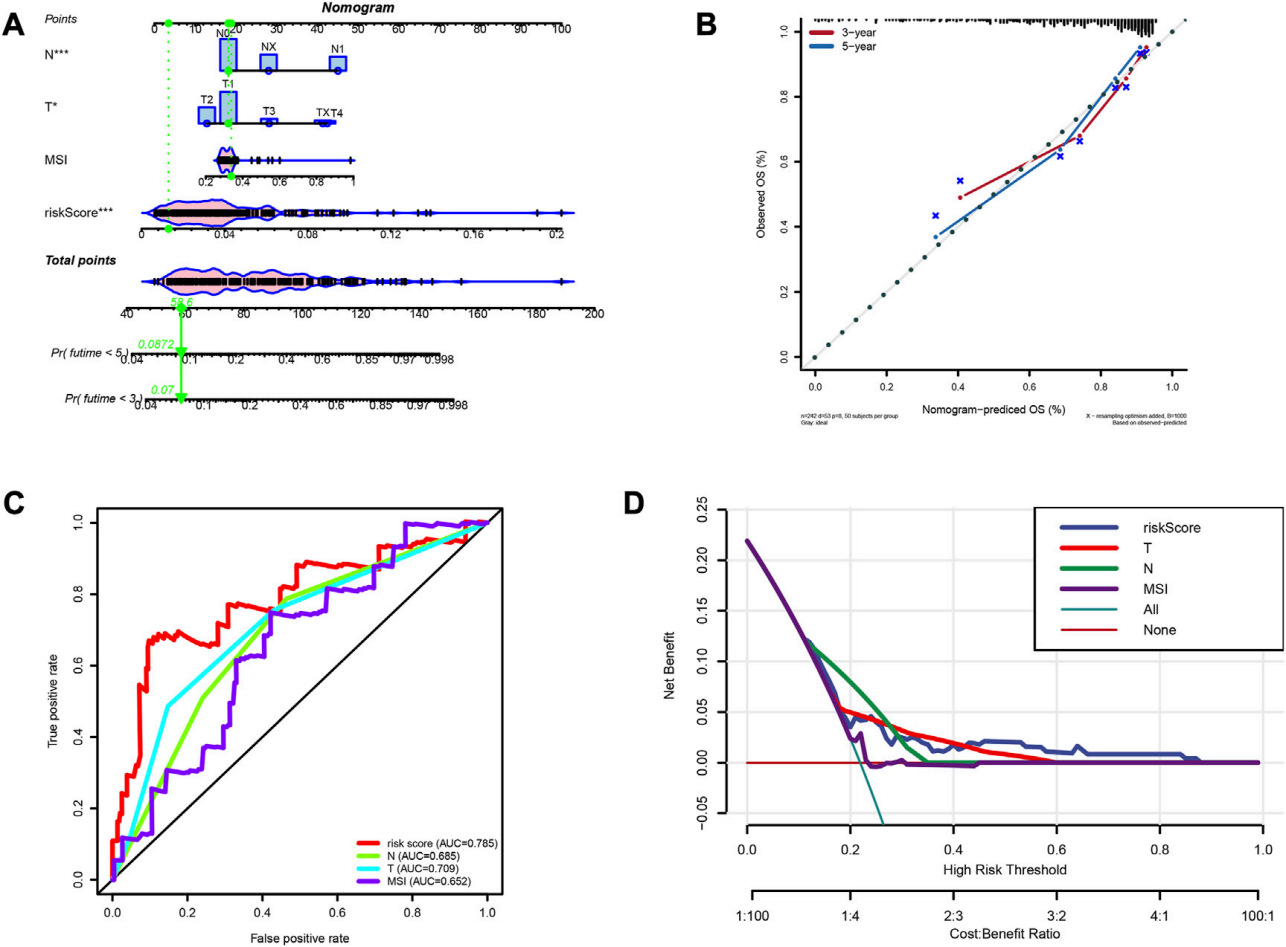
Construction of prognostic nomogram. **(A)** The nomogram predicts the probabilities of the 3- and 5-year outcome survival **(B)** The calibration plot for the nomogram predicts the probabilities of the 3- and 5-year outcome survival. **(C)** AUC values for factors in the nomogram. **(D)** The decision curve for the nomogram.

A nomogram was constructed to predict survivals after 3 and 5 years (Figure 9A). The calibration curve validated that the nomogram had a good accuracy in predicting patient survivals (Figure 9B). ROC and DCA showed that the risk score had a better clinical efficacy compared to several other characteristics (Figure 9C, D).

## Discussion

In the immune system, lncRNAs regulate gene expression and play a key role in tumorigenesis as well as progression (Li et al., 2020). In addition to serving as prognostic markers, IRLs may also serve as therapeutic targets for cancers (De Felice et al., 2018). lnc-INSR suppresses the immune microenvironment by regulating the differentiation of Treg cells (Liu et al., 2020), thereby promoting tumor growth. In diffused B-cell lymphoma, ncRNASNHG14 promotes immune escape by regulating immune checkpoints (Zhao et al., 2019). Oxidative stress can lead to inflammatory pathways through which normal cells are converted into tumor cells, and studies have shown that oxidative stress plays an important role in the progression of CC (18, 19). However, the exact role of IRLs in the prognosis of CC and their associations with oxidative stress remain unclear. In this study, we identified four lncRNAs and construct an immune risk scoring system. TFAP2A-AS1 is a tumor suppressor of breast cancer as it competes for miR-933, thereby releasing SMAD2 (Zhou et al., 2019). AL133215.2 is identified and used to construct a prognostic model for CC (Chen et al., 2020). LINC02078 and AP000911.1 have not yet been reported in this context. Next, we will discuss the potential utility of risk modeling as a new immunotherapeutic tool and analyze the relationship between risk modeling and oxidative stress.

The risk model was stratified into two groups based on risk level: low-risk and high-risk model, which was associated with oxidative-stress-related genes SOD2 and OGG1. SOD2 plays an important role in vascular oxidative stress (Dikalova et al., 2017), and OGG1 acts as a DNA repair enzyme that can counteract DNA damage caused by oxidative stress (Li et al., 2018). Patients in the low-risk group had a longer outcome survival, as well as a higher MSI and immunogenicity, who were also more suitable for anti-PD-1 therapies. Additionally, we also screened for drugs that showed a great sensitivity among low-risk patients, including bleomycin A2, dasatinib, afatinib, dinaciclib, and pelitinib. Risk scores were compared to other clinical characteristics, which were found to be independent risk factors. In a ROC curve analysis, the AUC value of the risk model was significantly higher than that of other characteristics, suggesting that risks were a better predictor of patient prognosis. A nomogram was constructed to predict patient survival after 1, 3, and 5 years. Importantly, we validated the expression of lncRNAs identified using the model through qRT-PCR and functionally validated one of the genes. This, to some extent, demonstrated the prognostic value of the risk model.

To evaluate the efficiency of risks in immunotherapies, the immunogenicity of the tumor microenvironment needs to be investigated (Gasser et al., 2017). TMB is a biomarker of immunotherapeutic response (Hellmann et al., 2018; Samstein et al., 2019), the higher the TMB is, the greater the benefit of immunotherapies will be. MSI is a major predictor of immunotherapeutic sensitivity; tumors with a high MSI (MSI-H) can be better treated with ICIs (Baretti and Le, 2018). HRD induces genomic instability and increases immunogenicity for patients with tumors, thereby leading to an increased response to ICIs (Dai et al., 2018). In this study, we found that risk grouping was significantly correlated with MSI grouping; the high-risk group had a higher MSI value. However, since microsatellite instability status does not effectively represent the potential benefit of immunotherapies (Camidge et al., 2019), other methods of evaluation need to be developed.

Mutations in certain genes are closely associated with immunotherapies, such as TP53, along with their associated co-mutations that can increase the expression of TMB and immune checkpoints, thereby affecting patients’ response to immunotherapies (Assoun et al., 2019). Of the 14 genes identified that showed mutational differences between the high- and low-risk group, 12 showed higher mutations in low-risk patients. AHNAK2 and BRCA1 are involved in the regulation of the immune system (Ju et al., 2020; Xie et al., 2020). Mutations in BRCA1, a homologous repair gene, can affect the efficacy of immunotherapies (Fumet et al., 2020).

Infiltration of immune cells can be used to predict the response to cancer immunotherapies (Junttila and de Sauvage, 2013). We used XCELL, EPIC and CIBERSORT algorithms to estimate the relationship between risks and immune cells, and the correlations between neutrophils and risks were positive. Neutrophils are early infiltrative inflammatory cells that enable tumor cells to escape immune surveillance (Fetz et al., 2021). A comparison between immune cells of the high- and low-risk group was analyzed with ssGSEA, which showed that the high-risk group had a higher neutrophil infiltration, while the low-risk group had a higher infiltration of NK-CD56 bright cells, NK cells and pDCs. The activation of plasmacytoid dendritic cells (pDCs) can induce T-cell activation or tolerance; the NK CD56 bright cells can be used as antitumor effectors in cancer immunotherapies (Wagner et al., 2017). From the above results, the low-risk group seemed to have a better immunogenicity. Combined with the results of the comparison of immune datasets, we speculated that patients in the low-risk group might respond better to immunotherapies.

Dinaciclib is an effective anti-PD1 inhibitor that induces immunogenic cell deaths (Hossain et al., 2018). Dasatinib, combined with low-intensity chemotherapies, is effective in Philadelphia-positive acute lymphoblastic leukemia (Rousselot et al., 2016). Obatoclax improves the response of patients with bladder cancer to cisplatin chemotherapies and their treatment outcomes (Steele et al., 2019). Although the role of these drugs in CC is rarely reported, in our study, by analyzing their potential efficacy in patients carrying PIK3CA mutations, we speculated that several drugs, including dasatinib, dinaciclib, and obatoclax, might show better efficacies in the low-risk group of patients. This could provide targeted therapy options for low-risk patients.

Our study innovatively identified and validated four IRLs for CC, uncovered immune-associated risk models for predicting clinical outcomes, and established links to oxidative stress. The key features we selected may define a new therapeutic strategy that will serve as new immune biomarkers for future CC immunotherapy. Meanwhile, in the risk model, a significant increase of the inflammatory response and oxidative phosphorylation was observed in the high-risk group of patients, suggesting that both inflammation and oxidative stress could lead to increased risks. AL133215.2 was lowly expressed in high-risk patients, therefore, the oxidative-stress-related pathways were significantly enriched in the AL133215.2 low-expression group.

## Conclusion

In conclusion, we developed a prognostic risk model by identifying IRLs and explored the associations between the risk model and oxidative stress. In actual clinical practice, we can perform transcriptome sequencing of 4 IRLs from patients, assess the risk scores and stratification of patients based on risk modeling formulas, and make comprehensive judgments on prognosis in conjunction with the clinical characteristics of patients. We can also propose the appropriate treatment plan according to the patient’s risk stratification. However, there are limitations to our study, including insufficient sample size, limited generalisability due to lack of information on patient treatment and long-term follow-up, and lack of more in-depth mechanistic studies. These limitations may affect the interpretation of our findings, but they do not negate the reliability of our study.

## Data Availability

The original contributions presented in the study are included in the article/Supplementary Material, further inquiries can be directed to the corresponding author.

## Abbreviations

CTRP2.0, Cancer Therapeutics Response Portal 2.0; CC, cervical cancer; CCLE, Cancer Cell Line Encyclopedia; CCK-8, Cell counting kit-8; CNV, copy number variation; DCA, decision curve analysis; GSVA, Gene set variation analysis; GSEA, Gene set enrichment analysis; HRD, homologous recombination deficiency; HPV, human papillomavirus; IRLs, immune-related lncRNAs; lncRNAs, long-stranded non-coding RNAs; MSI, microsatellite instability; PRISM, Profiling Relative Inhibition Simultaneously in Mixtures; ROC, receiver operating characteristic; SNP, single nucleotide polymorphism; TCGA, The Cancer Genome Atlas, lasso, least absolute shrinkage and selection operator; TPM, transcripts per kilobase million; TMB, Tumor mutational burden.
