# Supplementary material for: The construction of a prognostic model of cervical cancer based on four immune-related LncRNAs and an exploration of the correlations between the model and oxidative stress
Source: Front Pharmacol. 2023 Sep 21;14:1234181. doi: 10.3389/fphar.2023.1234181 (PMC10551162; doi:10.3389/fphar.2023.1234181)
Supplement: Supplementary file 3 [file Table1.DOCX]

**TFAP2A-AS1** (FORWARD: GGATCGGCGTGAACGGATATGC; REVERSE: TCTCCTCCTCCACCTCCTCTCC)；

**AP000911.1** (FORWARD: GTGCCACCTTTCCCTTCCTCTTTC ; REVERSE: TCCTTCACCATCCACGCTCCTC)；

**AL133215.2** (FORWARD: AACAGGAAATCTAGGAACCGAGAACAC; REVERSE: CCAAGGACCAGGGATTACTCACAAC)；

**LINC02078** (FORWARD: GGTGCTCCTGGGTGGGTCTC ; REVERSE: TCAGGGCAGGTGGCAAGTAGG)
